# Surgery

**Published:** 1908-06

**Authors:** E. H. E. Stack


### SURGERY.

**Brain Surgery.**—During the last few years advance has been made in greater accuracy of diagnosis, larger number of cases operated on, and higher percentage of successes.

The introduction of large osteoplastic flaps, and systematic operations for relief of symptoms without removal of the disease, are the two chief innovations.

*Injuries.*—Trauma at birth, giving rise to asphyxia; a bulging fontanelle without pulsation, convulsions and hemiplegia, and other signs of compression, with usually a rise of temperature, have had bone turned down and extravasated blood let out, with a large percentage of successful results.[1]

*Fractured base.*—Cushing[2] relates two cases with compression symptoms, in which, a few days after the injury, clots were let out and a drain put in with recovery. During the last year he had performed this operation almost invariably after fracture of the base, because nearly all the symptoms are due to intra-cranial pressure. It gives the surgeon a definite way of attacking what had been before considered pretty hopeless cases. Most neurologists believed that this operation, in these cases, is most apt to do away with the distressing features of post-traumatic neuroses.

Dennis[3] points out that as the brain is incompressible and the cranium inexpansile, hemorrhage, or other foreign body, must find room at the expense of the blood and lymphatics. He therefore advises operation always for pressure with coma, and for pressure without coma if the symptoms are progressive.

He relates a case of a child which fell from the third floor to the pavement, and had coma and hemiplegia from a depression of the skull in the motor area, and was relieved of both conditions immediately by manipulation, raising the bone. The writer saw two cases of this sort in children of two years or under at the Bristol Royal Infirmary, where pond depressions, larger than a teaspoon, were elevated by him in a similar way, just as one can raise the dent out of a felt hat, the bone springing to its level again with a slight noise.

Intermittent headache, paralysis, and even mania after an injury, is suggestive of arachnoid hemorrhage, and is curable by operation. Lumbar puncture will tell the nature of the fluid in intradural or arachnoid space, but not differentiate them; it is of great use, occasionally, in the diagnosis of hemorrhage in fractured base.[1] In extradural hemorrhage the fluid, of course, will be clear.

*Traumatic encephalocele,* according to Ballance, demands that the bone be cut away till the tumour pulsates freely, after which, if all goes well, it returns gradually to the cranial cavity.

*Meningocele and Encephalocele.*—Operation is contra-indicated when the soft parts are deficient as well as the bone, as in a toad monster, to take an extreme case: also when in the occipital region there is, in addition, deficiency in the upper spinal region; and thirdly, when there is as well hydrocephalus.

In other cases the only treatment is with the knife; if there be brain also in the tumour it is no matter, it should be clamped and turned in or tied off. A stop-gap of some foreign body may have to be used to supply the want of bone.[5]

*Hydrocephalus.*—In spite of numerous procedures, there is nothing reliable at present in the way of treatment.[5]

*Meningitis* is beginning to come a little more within the reach of surgery.

*Traumatic.*—Pus between the dura and bone, from scalp wounds, is much rarer than it used to be when Pott first described his puffy swelling: "It is very important to trephine early in these cases, should any symptoms arise in the second week."[4] The dura is very resistant, and extra-dural abscess, from this or other causes, may be present weeks without giving rise to meningitis.[1]

*From bone or sinus infection.*—The treatment in ear cases is well recognised. Frontal and ethmoidal suppuration should be treated in the same way that temporal is, by complete ablation.[1] If meningeal symptoms continue after a mastoid operation, the dura should be opened.

Hinsberg refers to several cases of apparently hopeless *suppurative meningitis* cured by drainage in the posterior fossa on both sides.[1]

*Tubercular Meningitis.*—Ballance[1] says: "Until more complete operations have been performed in early stages of tuberculous meningitis (*i.e.* draining the arachnoid space and sylvian lakes on both sides), we cannot say whether the disease is likely to be modified in the same favourable manner by operation as tuberculous peritonitis."

*Abscess.*—These have been reported occasionally as cured without operation; if so, they were probably tubercular.

Ballance lays stress on following an abscess by its track, whether traumatic or due to bone disease. If they can be opened through their stalk, it prevents closing of the sinus, and also hernia, besides lessening the chance of infecting the adjacent meninges.[6] To explore for an abscess, he advocates a long, sharp-pointed, narrow knife, as much better than trocar, grooved needle, etc.

*Tumour.*—Whether a tumour should be partially removed when total ablation is impossible, on the assumption that they grow more slowly after such treatment, is a subject of argument. Ballance says yes, Spiller[7] no. About tumours there is one opinion in which all writers seem to be in accord, that is that where a tumour cannot be removed—either on account of its size, or more often because the localising symptoms are absent or inefficient—then a palliative procedure should be done. This will relieve headache, vertigo, nausea, vomiting, and, most important of all, optic neuritis. This latter is really a congestion, not an inflammation; if relief of tension comes soon enough, it will clear up almost at once, and so save the patient from going blind.

Compression of the brain, therefore, may be operated on in two kinds of cases. Just as a band divided in acute intestinal obstruction gives relief, so will trephining in acute compression from a ruptured meningeal artery; and as colotomy relieves chronic obstruction, so decompression will do the same for the brain.

The best spot to choose for relief of tension depends on whether an attempt is to be made at the same time to remove the cause.[8] If the situation is unknown, then a region well covered by muscle, such as the temporal or occipital, is chosen, because after an area of bone has been removed the muscle will control the size of the curative hernia, and avoid fungation ending in meningitis. Some remove portions of dura mater. Frazier[7] is strongly opposed to this, and quotes many cases to show that it is unnecessary.

As a practical precaution,[9] lumbar puncture, to diminish tension, should be done before opening the dura in any compression case, as it facilitates exploration, and saves the brain from damage by bulging during the operation.

*Technique.*—There remains only to be said a few words on this subject. Many cases are best operated on in two stages; it lessens the shock, and the absence of meningeal bleeding is a great assistance at the second operation. Cushing[10] has also discovered that a tumour may be removed with almost no pain without anæsthesia, which may prove a method of great value in the future.

Bergmann[11] sums up the methods of opening the skull. He advocates, as most do now, the osteoplastic flap, made either by Wagner's plan, chiselling through the bone; or Doyen, with his burrs and saws; or perhaps, best of all, Obalinski's, with Gigli's saw and trephine holes. Sudek's trephine drill is another good, rapid method.

When bone has had to be taken away, and it is advisable, if possible, to close the gap again, the best method seems to be that of Müller and König, who elevate a living flap of bone—the outer table only—and slide it across with a periosteal hinge to cover the space.

The greatest advance of all is in the size of the flaps, often several square inches in area, which is useful to the surgeon for many reasons, not the least being that accurate topography is now almost unnecessary.

E. H. E. Stack.

REFERENCES.

1 C. A. Ballance, "Some Points in the Surgery of the Brain," 1907.
2 *Johns Hopkins Hosp. Bull.*, 1908, xix. 48.
3 *Med. News*, 1903, lxxxii. 535.
4 Jacobson and Rowlands, "The Operations of Surgery," 5th Ed., 1907, i. 252.
5 Bergmann, "System of Practical Surgery," i. 170 *et seq.*
6 Ballance, Op. cit., p. 141 *et seq.*
7 Spiller and Frazier, *Univ. Penna. M. Bull.*, 1906-7, xix. 146.
8 Cushing, *Surg., Gynce. and Obst.*, 1905, i. 297.
9 Ballance, Op. cit., p. 199.
10 *J. Am. M. Ass.*, 1908, l. 847.
11 Bergmann, Op. cit., p. 367 *et seq.*

